# Neonatal Diet and Gut Microbiome Development After C-Section During the First Three Months After Birth: A Systematic Review

**DOI:** 10.3389/fnut.2022.941549

**Published:** 2022-07-26

**Authors:** Eliska Pivrncova, Iva Kotaskova, Vojtech Thon

**Affiliations:** RECETOX, Faculty of Science, Masaryk University, Brno, Czech Republic

**Keywords:** breastfeeding, delivery mode, C-section, infant, nutrition, microbiome, bacteria

## Abstract

**Background:**

Cesarean section (C-section) delivery imprints fundamentally on the gut microbiota composition with potential health consequences. With the increasing incidence of C-sections worldwide, there is a need for precise characterization of neonatal gut microbiota to understand how to restore microbial imbalance after C-section. After birth, gut microbiota development is shaped by various factors, especially the infant’s diet and antibiotic exposure. Concerning diet, current research has proposed that breastfeeding can restore the characteristic gut microbiome after C-section.

**Objectives:**

In this systematic review, we provide a comprehensive summary of the current literature on the effect of breastfeeding on gut microbiota development after C-section delivery in the first 3 months of life.

**Methods:**

The retrieved data from PubMed, Scopus, and Web of Science were evaluated according to the PICO/PECO strategy. Quality assessment was conducted by the Newcastle–Ottawa Scale.

**Results:**

After critical selection, we identified 14 out of 4,628 studies for the evaluation of the impact of the diet after C-section delivery. The results demonstrate consistent evidence that C-section and affiliated intrapartum antibiotic exposure affect Bacteroidetes abundance and the incapacity of breastfeeding to reverse their reduction. Furthermore, exclusive breastfeeding shows a positive effect on Actinobacteria and Bifidobacteria restoration over the 3 months after birth. None of the included studies detected any significant changes in *Lactobacillus* abundance in breastfed infants after C-section.

**Conclusion:**

C-section and intrapartum antibiotic exposure influence an infant’s gut microbiota by depletion of Bacteroides, regardless of the infant’s diet in the first 3 months of life. Even though breastfeeding increases the presence of Bifidobacteria, further research with proper feeding classification is needed to prove the restoration effect on some taxa in infants after C-section.

**Systematic Review Registration:**

[www.crd.york.ac.uk/prospero/], identifier [CRD42021287672].

## Introduction

The acquisition and development of the intestinal microbiome is a dynamic process shaped by various factors. Starting at birth, the microbial community evolves into a stable and complex microbiome, resembling an adult one by the age of 3 (1–3). Despite current discussion about prenatal contact with microbes *in utero*, the first major transition of gut microbiota occurs during birth (4, 5). Vaginal delivery (VD) is a natural process where neonates are exposed to bacteria in the maternal birth canal. When either the mother or baby is at risk, cesarean section (C-section) delivery is necessary. Rates of this surgical procedure continue to rise globally, now accounting for more than one in five (21%) of all childbirths, depending on the access of the procedure (6). In contrast to vaginal birth, cesarean section delivery (CS) transmits distinguished gut microbiota from the maternal skin and hospital environment. Moreover, CS neonates are also indirectly exposed to antibiotics (ATB) through intrapartum prophylaxis (IAP). These early-life exposures are associated with reduced microbiota diversity and altered taxonomic distribution of gut microbiota (7, 8). Commonly observed patterns are *Bifidobacterium* and *Bacteroides* depletion and *Enterococcus*, *Staphylococcus*, *Streptococcus*, *Enterobacter*, and *Clostridioides* increases (9–11). These perturbations potentially lead to long-term effects on health in childhood and later in life (12–14).

Following birth, the microbiome is shaped by complex interactions between the mother, the infant, and their environment (5, 15). One of the major determinants of establishing a healthy gut microbiome is the neonatal diet (16, 17). Breastmilk provides optimal nutrition not only for the infant but also for the intestinal microbial community. It is a complex fluid of nutrients, bioactive compounds, and bacteria that support healthy infant development (18). Nevertheless, breastfeeding is not successfully initiated in the case of every infant. One of the reasons is the mode of delivery. CS influences lactogenesis, delays the establishment of breastfeeding, and discourages the process from the beginning (19, 20). Donor milk is an effective substitute in preterm infants, while formula feeding is common practice in healthy term infants. Although infant formula has a standardized composition, some nutrients, bioactive compounds, or live cells cannot be added to it due to negative interactions, short shelf life, bioavailability, or excessive production costs (21, 22). This, in turn, affects the neonatal gut microbiome. Several studies characterize the microbiome of formula-fed infants as distinct in wider microbiota diversity, resembling the weaning profile. The percentage of *Bifidobacterium* and *Bacteroides* is downregulated, *Clostridioides*, *Streptococcus*, and *Enterococcus* are increased; opportunistic pathogens are present (23, 24). The gut microbiome profile after formula feeding notably resembles the profile reported in CS infants; therefore, there is a question to what extent the gut microbiome perturbations in CS infants are a result of birth exposure or the following diet. Moreover, some recent studies declare that breastfeeding restores the distinctive gut microbiome after CS delivery (25–27).

To our knowledge, previous systematic reviews have assessed the impact of the delivery mode or the impact of feeding mode on the infant gut microbiome independently (7, 28, 29), with one exception emphasizing breastfeeding (30). Accordingly, our review aims to systematically assess existing publications describing the influence of delivery mode together with neonatal diet on the gut microbiome. We considered the optimal period to assess the effect of diet on the gut microbiome of CS infants from the first week to three months. The interval ensures that initiated lactation influences the infant’s gut microbiome and that complementary feeding is not implemented into the infant’s diet and impacts the results. Thus, in our systematic review, we compare the microbiome profiles of breastfed CS and VD infants from the first week to three months after birth. Furthermore, we describe the gut microbiome profile in formula-fed CS infants if they were stratified in studies included in our systematic review. This allows us to evaluate the effect of breastfeeding on the CS infant microbiome.

## Materials and Methods

This systematic literature review was registered with the International Prospective Register of Systematic Reviews (PROSPERO registration No. CRD42021287672). The research question “Does breastfeeding restore the gut microbiome of healthy full-term infants born by C-section in the first 3 months of life compared to vaginally delivered infants?” was defined using the PICO/PECO strategy (for PICO/PECO elements, see Table 1).

**TABLE 1:** PICO/PECO elements definition.

Variable	Definition
Population	Healthy term infants (≥ 36 weeks)
Intervention/ exposure	Type of feeding (breastfeeding/formula-feeding) in C-section deliveredes
Comparison	The microbiome of breastfed vaginally deliveredes
Outcome	Gut microbiome composition in the first three months of life

### Study Identification

We used PubMed, Scopus, and Web of Science to identify relevant articles on the effect of feeding practice on the gut microbiome in the first month after birth. We searched for the following MeSH terms: “(nutrition OR diet) AND (infant OR neonate OR newborn) AND (gut OR intestinal) AND (microbiome OR microbiota OR bacteria OR microbial)”. Only studies published before 1 September 2021 were included.

### Study Selection and Inclusion and Exclusion Criteria

Two of the authors (EP and IK) separately searched the databases using the key search string. Once duplicates were removed, EP and IK independently reviewed the remaining articles by title and abstract to either retain or discard them according to the inclusion and exclusion criteria. This systematic review only includes observational studies of various designs (e.g., prospective, retrospective, cross-sectional, cohort, or case–control). The following studies were excluded: (1) non-human and *in vitro* studies; (2) studies not in English; (3) studies on preterm infants (<36 weeks), infants with very low birth weight (<1,500 g) or infants suffering from any disease/disorder; (4) studies focusing exclusively on supplementation, probiotics, and prebiotics; (5) studies with results not considering the mode of delivery and feeding together; (6) studies without control groups (breastfed vaginally delivered infants); (7) studies using samples collected only within the first week after birth or after 3 months post-partum; and (8) studies not empowering culture-independent molecular techniques for bacterial detection in stool samples. In the case of uncertainties, the full text was investigated. Any disagreements were resolved through discussion. In addition to the database searches, forward searches of included studies and relevant reviews were performed to identify additional sources.

### Data Collection

After the data extraction study, details were tabulated as follows: study overview (author identification, year of publication, country of origin, population size), subject characteristic (mode of delivery, feeding type), study design (sampling time points, methodology used, amplified region/primers, platform), and key findings (changes in diversity and taxonomical changes). If necessary, authors were additionally contacted to clarify unclear details.

### Strategy for Data Synthesis and Quality Assessment

For data synthesis, a descriptive synthesis was applied. Where available and comparable, data on diversity (OTU/ASV counts and Shannon index) were extracted for particular groups. The quality assessment was performed based on Newcastle–Ottawa Scale quality assessment (31). Points were assigned on a nine-point scale for quality factors including (i) comparability of exposed and non-exposed groups; (ii) evidence of microbiome assessment prior to exposure; (iii) record of diet; (iv) confounding factors; and (v) statistical analysis (Supplementary Table 1). The point for representativeness of the exposed cohort was allocated if the cohort of breastfed CS infants was ≥10.

## Results

### Study Selection and Characteristics

**FIGURE 1:**
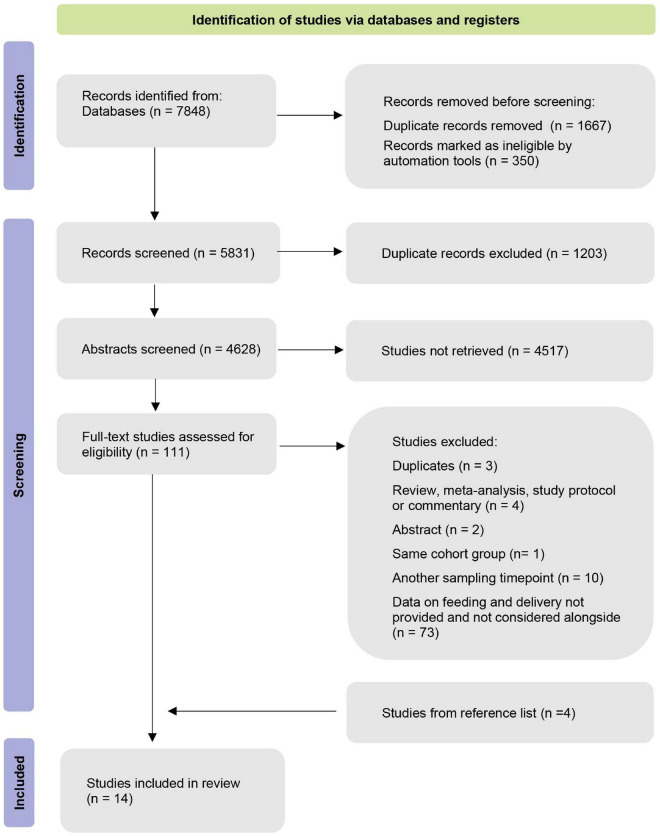
Identification of studies via databases and registers.

Our database search yielded 7,848 publications. In total, 1,667 duplicates were removed after the duplicate search in Mendeley and Excel. Afterward, we screened the remaining 5,831 records based on their title and abstract. Four additional studies were identified through forward searches in the reference list. Altogether, we selected 111 studies for full-text screening. Finally, 98 studies were excluded based on the defined inclusion/exclusion criteria (Figure 1). The remaining 14 manuscripts were eligible for our systematic review.

### Study Characteristics

Overall, 14 selected manuscripts (Table 2) comprise a cohort of 3,091 infants: 626 breastfed CS infants, 134 non-breastfed CS infants, and 1,187 breastfed VD infants. All studies were conducted in high-income countries: six studies from North America (32–37), four from Europe (38–41), three from Asia (26, 27, 42), and one from South America (43). Selected studies were published in the last 10 years between 2014 and 2021. The majority of included studies provided details on study exclusion criteria, covering, for instance, ATB use in postnatal life. Two studies analyzed the effect of intrapartum antibiotic prophylaxis (IAP) in VD, elective, and acute CS delivery (33, 35). Two studies investigated the effect of a mother’s secretor status (40, 43).

**TABLE 2:** Characteristics of the studies. CS-EBF indicates C-section exclusively breastfed infants, CS-MBF indicates C-section mix-fed infants, CS-NBF indicates C-section non-breastfed infants, and VB-BF indicates vaginally delivered breastfed infants.

Author	Country	Sample size					Stool collection	Microbiome analysis method
				
		(n)	CS-EBF	CS-MBF	CS-NBF	VB-BF		
Fehr et al. (32)	Canada	591	NS	NS		NS	3 months and 1 year	Illumina MiSeq
Chen et al. (33)	Canada	1654	198	124	81	727	3 months	Illumina MiSeq, qPCR
Azad et al. (34)	Canada	198	22	34		55	3 months and 1 year	Illumina MiSeq
Brumbaugh et al. (35)	US	23		12		11	2 weeks and 6 weeks	Illumina MiSeq
Bokulich et al. (36)	US	43	7*		7	15	1-12 months and 14,16,18,20,24 months	Illumina MiSeq
Madan et al. (37)	United States	102	20	10	2	50	6 weeks	Illumina MiSeq
González et al. (38)	Spain	124	18	18*		56	1 month	qPCR
Hill et al. (39)	Ireland	219	111			102	1, 4, 8, 24 weeks	Illumina MiSeq
Korpela et al. (40)	Finland	91	23*			68	3 days and 3 months	Illumina MiSeq or HiSeq
Jakobsson et al. (41)	Sweden	24	9			15	1 week and 1,3,6,12,24 months	454 GS FLX
Liu et al. (42)	China	94	24		10	43	6 weeks	Illumina HiSeq
Akagawa et al. (27)	Japan	36		10	6	10	4 days and 1 month	Ion PGM Sequencer and Ion 318 Chip
Guo et al. (26)	China	41	7		10	14	1,3,7 days and 1,3,6 months	Illumina MiSeq
Tonon et al. (43)	Brazil	54	27			21	1 month	Illumina MiSeq, qPCR

*Refer to a group with a not clearly specified diet.

Fecal samples were collected at various time points during the follow-up, except for five studies with only one time point of sample collection (33, 37, 38, 42, 43). For the evaluation of fecal samples, three studies applied the qPCR technique (33, 38, 43), 10 studies used Illumina MiSeq, and one used the Illumina HiSeq NGS platform, predominantly targeting V4 and V3–V4 regions. The remaining two studies are Ion Torrent and 454 sequencing (27, 41).

The feeding mode was categorized into diverse groups and the approach in the definition of breastfeeding differed between studies. In total, nine studies described the group of exclusively breastfed infants. The rest of the studies defined breastfed group as a group fed with breast milk by more than 80% (27) or as a mix of breastfed and partially breastfed infants (35, 40).

The quality assessment (Newcastle–Ottawa Scale) of selected studies is presented in Supplementary Table 1. A total of seven studies were awarded six points, and six studies were awarded more than seven points. Within the very strict inclusion criteria in our systematic review, no studies obtained five points or less. Also, no study achieved a maximum of nine points.

### Data Evaluation

#### Gut Microbiome in the First 2 Weeks of an Infant’s Life

Bacteroidetes (35, 41) and *Bacteroides* (39) were significantly increased in VD infants compared to CS infants in all studies analyzing breastfed infants only. Only two studies highlighted a higher relative proportion of Firmicutes dominating in CS infants (39, 41). Hill et al. (39) showed a significant decrease in Actinobacteria and pointed to differences in the proportion of Bifidobacteria in the first week between breastfed CS and VD infants (19 vs. 48%). However, the difference was not significant due to the high inter-individual variation between infants at this timepoint. Only one study pointed to a significant difference in the Shannon index between CS and VD breastfed groups (39), whereas other studies in the first 2 weeks did not find any significant difference in microbial diversity.

Only Guo et al. (26) described the effect of formula feeding in CS infants for the first week of the infant’s life. This study did not identify any differences in the gut microbiome pattern.

#### Gut Microbiome in the First Month of the Infant’s Life

In the first month, CS breastfed infants maintained a characteristic significant reduction of Bacteroidetes (35, 43) and *Bacteroides* (27, 35–37, 39, 43) compared to VD breastfed infants. Some studies described Bacteroides as the only increased genus in CS exclusively breastfed infants at 1 month (27, 36, 37). Nevertheless, filling the void left by *Bacteroides*, some studies underlined other increased genera in breastfed CS infants, more specifically significantly increased *Akkermansia* (the most abundant genus of Verrucomicrobia) (43), *Kluyvera* (usually the most abundant genus of Proteobacteria) (43), and *Enterococcus* (41). Liu et al. (42) reported an increased *Enterococcus* and *Veillonella* abundance in mix-fed CS infants; however, they did not observe any significant difference in gut microbiota in exclusively breastfed CS infants compared to exclusively breastfed VD. Studies applying either the qPCR technique (38, 43) or 16S rRNA sequencing (27, 35–37, 39, 41) did not reveal any significant differences in *Bifidobacterium* spp. abundance in breastfed CS and breastfed VD infants. Only Hill et al. (39) presented a significant difference in the relative proportion of Actinobacteria, yet the relative proportion of *Bifidobacterium* was insignificant at all time points. Data presented by Gonzales et al. (38) showed an insignificantly lower abundance of *Bifidobacterium.*

The microbiome composition in formula-fed CS infants shifted from the first week to the first month and resulted in a significantly lower abundance of Bifidobacteria than breastfed CS and VD infants in the first month (26). This is in agreement with data presented by Bokulich et al. (36). Gonzales et al. (38) did not reveal any significant differences in bacterial groups in formula-fed CS and VD infants by qPCR. However, the values of *Enterococcus* abundance in CS formula-fed infants were the highest compared to other feeding/delivery groups. Bokulich et al. (38) showed a higher abundance of Firmicutes, Clostridiales, and Proteobacteria. Liu et al. (40) reported that the absence of exclusive breastfeeding significantly increased *Enterococcus*, *Veillonella*, and *Faecalibacterium* abundance; two studies did not describe differences in any taxa, except *Bacteroides* in formula-fed CS infants compared to other feeding types. However, these studies had a very small sample size of formula-fed CS infants (27, 37). Concerning *Bacteroides*, the abundance remains lower in studies including breastfed and formula-fed CS infants, regardless of feeding type (27, 36, 38).

#### Gut Microbiome in Three Months of an Infant’s Life

In the third month of life, in fants born by CS were still characterized by a significant reduction in Bacteroidetes (33, 34, 40, 41) and *Bacteroides* (32–34, 36, 40, 41). Korpela et al. (40) reported significantly reduced Actinobacteria. Regarding Bifidobacteria, Chen et al. (31) revealed differences between exclusively breastfed infants delivered by elective CS and emergency CS. However, CS breastfed infants compared to VD infants did not significantly differ in most of the 16S rDNA amplicon sequencing (32, 34, 40, 41) or qPCR studies (33). Moreover, Chen et al. (33) described a decreased abundance of *Clostridium* and *Enterococcu*s genus in CS compared to VD breastfed infants. Azad et al. (34) reported an increase in *Clostridium* taxa in exclusively breastfed infants after emergency CS but not after elective CS. Fehr et al. (32) presented significant differences between exclusively breastfed CS and VD infants in *Bacteroides uniformis*, *Enterococcus*, and *Veillonella dispar*. Korpela et al. (40) highlighted the remaining increased abundance of *Verrucomicrobia* from one month in CS breastfed infants of secretor mothers; two studies describing a similar trend in the first month did not find any significant difference at 3 months (36, 41).

When exclusive breastfeeding is reduced to partial breastfeeding, the gut microbiota profile of CS infants differed in Bacteroides, *Clostridium paraputrificum*, *Enterococcus*, *Lachnospiraceae*, and *Veillonella dispar* abundance (32). Azad et al. (34) pointed to a significantly lower abundance of Bacteroidetes and a higher abundance of Clostridiales in partially breastfed infants. Bokulich et al. (36) characterized the gut microbiota of formula-fed infants at 3 months by increased *Ruminococcaceae* and *Lachnospiraceae*. Absolute quantification of *Bifidobacterium* by qPCR revealed lower absolute quantities in CS formula-fed infants than VD breastfed infants (33). Bokulich et al. (36) showed an increased abundance of Proteobacteria in gut microbiota both in CS formula-fed and CS exclusively breastfed infants.

The main findings of significant differences in the gut bacterial abundance within the first 3 months of an infant’s life are presented in Table 3.

**TABLE 3:** Main findings of significant differences in the gut bacterial abundance of exclusively breastfed, mix-fed, and non-breastfed CS infants compared to breastfed VD infants.

	Exclusively breastfed cs infants		References	Mix-fed CS infants		References	Non-breastfed		References
**1-2 WEEKS**	**Bacteroidetes**	**↓**	(41)	**Bacteroidetes**	**↓**	(35)			
	*Bacteroides*	**↓**	(39, 41)	*Bacteroides*	**↓**	(35)			
	**Actinobacteria**	**↓**	(39)						
	**Firmicutes**	**↑**	(39)						
	*Veillonella*	**↑**	(41)						
**4-6 WEEKS**	**Bacteroidetes**	**↓**	(43)	**Bacteroidetes**	**↓**	(35)			
	*Bacteroides*	**↓**	(36, 37, 39, 43)	*Bacteroides*	**↓**	(27)	*Bacteroides*	**↓**	(26, 36, 38)
	**Actinobacteria**	**↓**	(39)				**Actinobacteria**	**↓**	(36)
							*Bifidobacteria*	**↓**	(26, 36)
	**Firmicutes**	**↑**	(39)				**Firmicutes**	**↑**	(35)
							*Clostridiales*	**↑**	(35)
	*Enterococcus*	**↑**	(41)	Enterococcus	**↑**	(42)			
				*Veillonella*	**↑**	(42)			
				*Faecalibacterium*	**↑**	(42)			
	**Verrucomicrobia**	**↑**	(43)						
	*Akkermansia*	**↑**	(43)						
							**Proteobacteria**	**↑**	(35)
	*Kluyvera*	**↑**	(43)						
**3 MONTHS**	**Bacteroidetes**	**↓**	(33, 34, 41)	**Bacteroidetes**	**↓**	(40)			
	*Bacteroides*	**↓**	(32–34, 36, 41)	*Bacteroides*	**↓**	(32, 34)			
	*Bacteroides unifomis*	**↓**	(32)						
				*Parabacteroides*	**↓**	(40)			
				**Actinobacteria**	**↓**	(40)	**Actinobacteria**		
							*Varibaculum*	**↓**	(36)
							*Bifidobacterium*	**↓**	(33)
	**Firmicutes**	**↑**	(32)	**Firmicutes**	**↑**	(40)	**Firmicutes**	**↑**	(33, 36)
	*Clostridium*	**↑**	(33, 34)				*Clostridium*	**↑**	(33)
							*Clostridiales*	**↑**	(36)
				*Clostridium paraputrificum*	**↑**	(32)			
	*Enterococcus*	**↑**	(32, 33)	*Enterococcus*	**↑**	(32)	*Enterococcus*	**↑**	(33)
				*Lachnospiraceae*	**↑**	(32)			
	*Veillonella*	**↑**	(33)	*Veillonella*	**↑**	(33)	*Veilonella*	**↑**	(33, 35)
				*Veilonella dispar*	**↑**	(32)			
							*Lachnospiraceae*	**↑**	(35)
				**Verrucomicrobia**	**↑**	(40)			
	**Proteobacteria**	**↑**	(33)				**Proteobacteria**	**↑**	(33)

## Discussion

### Reduced Bacteroides Abundance in Cesarean Section Delivery Breastfed Infants

Our systematic review reports several findings in gut microbiome development in the first 3 months of an infant’s life. First, all studies presented data on the reduced relative abundance of *Bacteroides*. This concurs well with most of the studies presenting delayed colonization of Bacteroides as a fundamental characteristic of CS-delivered infants (10, 44, 45), indicating the essential role of attributes of cesarean delivery in the establishment of early gut microbiota. It is noted that *Bacteroides* and *Parabacteroides* are most frequently transmitted from the mother to neonates through vaginal birth (15, 46). In addition to that, *Bacteroides* depletion is associated with maternal IAP. The effect of IAP is supported by studies describing the administration of IAP in vaginal delivery, where a similar trend of *Bacteroides* reduction is reported (32, 33, 45, 47). Both maternal transmission and IAP cause substantially lower colonization of *Bacteroides* from the first day after birth in CS infants. This is in agreement with recent findings by Mitchell et al. (48). Interestingly, they emphasize the presence of reduced *Bacteroides* species during the first week of life, followed by the disappearance of Bacteroides species in the second week. With these results, they point to the stability of acquired microbiome composition after C-section. More specifically, *Bacteroides* stability could be shattered by the competition of co-occurred taxa present after initial birth seeding, by lack of supporting factors like breastfeeding, and by differences in diversity. In their study, CS infants were less likely to have *B. fragilis* or *B. thetaiotaomicron* than VD infants.

This shows the importance of *Bacteroides* in an infant’s health and the gut microbiome. Previous studies refer to the effect of *Bacteroides fragilis*, *Bacteroides vulgatus*, or *Bacteroides thetaiotaomicron* on the immune system by activating T-cell-dependent immune responses (49–52). *B. uniformis* is also involved in glycan metabolization and its abundance increases in response to breastfeeding. Martin et al. (44) showed a lower probability to detect *B. fragilis* and *B. uniformis* during the first 3 months of life in CS infants. In this systematic review, Fehr et al. (33) reported a significant reduction of *B. uniformis* in CS infants, not only at 3 months but also at 1 year.

There is no agreement on how long the reduction of *Bacteroides* lasts (5, 7). Our systematic review showed a significant decrease in *Bacteroides* within 3 months. However, the reduction is often traced to at least 6 months of age. In addition, some studies presented *Bacteroides* as the only differentially abundant genera persisting over 3 months in breastfed CS infants and even up to 1 year (6, 9, 36). Most importantly, our systematic review observed that even exclusive breastfeeding did not equalize the difference in *Bacteroides* abundance after initial birth seeding within 3 months. Compared to formula-fed CS infants, exclusive breastfeeding positively influences the abundance of *Bacteroides* in CS infants but still does not restore the significant difference. Thus, the length of breastfeeding accelerates *Bacteroides* recovery within the first year (34, 41).

### Effect of Breastfeeding on Bifidobacteria

Another widely discussed genus *Bifidobacterium* is regarded as a key intestinal taxon in early life. Bifidobacteria generate a low-pH environment, produce antimicrobial polysaccharides, and consequently influence the presence of other microbes (53, 54). A reduction in Bifidobacteria, related perturbations in interlinked microbes, and disruption of their functions at an early age are associated with immune and metabolic disorders (55–57). Regarding the Bifidobacteria depletion, the aberrant numbers have been described in CSes and after ATB treatment. All species and strains of *Bifidobacterium* are sensitive to IAP exposure (58–60). Stearns et al. (61) reported that every hour of IAP administration decreases the abundance of Bifidobacteria at 12 weeks. This is in agreement with the study of Chen et al. (33) who indicated a negative effect of IAP on VD exclusively breastfed infants at 3 months. The systematic review of the colonization pattern after CS delivery done by Rutayisire et al. (7) affirms the association between C-section and lower abundance of Bifidobacteria from birth to 3 months of life. Moreover, it is generally accepted by the scientific community that the genus *Bifidobacterium* dominates in the gut microbiota of VD breastfed infants (56) and that breastfeeding supports the growth and presence of Bifidobacteria (62). Therefore, it raises the question of how C-sections affect the abundance of Bifidobacteria in exclusively breastfed infants and if breastfeeding has the power to restore the effect of initial CS seeding and IAP on Bifidobacteria.

The evidence presented by our systematic review shows a significant reduction in the phylum level of Actinobacteria (39, 40) but an insignificant decrease in *Bifidobacterium* abundance in breastfed CS infants. This interpretation contrasts with studies not stratifying the feeding mode in C-section delivery. Moreover, the importance of breastfeeding is supported by the results in formula-fed CS infants, in which Bifidobacteria significantly decreased in abundance in the first month (36) and third month (33, 36).

The principal factor in the selective growth of intestinal *Bifidobacterium* spp. is human milk oligosaccharides (HMOs). HMOs are essential components of breast milk, which plays an important role in an infant’s gut microbiome development. They act as prebiotics, anti-adhesive, antimicrobial, and antibiofilm agents (63, 64). HMO concentrations vary widely between mothers and are associated with multiple factors (65). One characteristic is genetic secretor status, which determines the synthesis of fucosylated HMO absent or depleted in the milk of non-secretor mothers. These HMOs are degraded by enzymes possessed by strains of Bifidobacteria and *Bacteroides* (66, 67), microbes decreased in CS infants. In addition, the HMO profile and secretor status differ between populations (ranging from 65% to 98%) (68). Because of this, it is relevant to consider the secretor status in studies describing the effect of delivery and feeding mode on an infant’s gut microbiome. Korpela et al. (40) noted that CS intestinal microbiota may be detrimental if the infants are breastfed by a non-secretor mother.

In the same way, there is a need to consider geographical location in the comparison of results for genetic characteristics and also for cultural variations in feeding practices, hygiene, and lifestyle (69, 70). For instance, Princisval et al. (30) summarized the variations of proportion in Bifidobacteria favored in the northeast a south/southeast Asia, or *Bacteroides* more abundant in Central Europe. Half of the studies included in this systematic review represent CS infants from North America, only three studies were conducted in Asia, and one in South America. Given this, more studies are needed from various populations for a better understanding of the topic.

The intraindividual spectrum of HMOs in breast milk creates a stark contrast to the infant formula with few prebiotics. Even though the composition of infant formula evolved immensely over the last decade, it is not feasible to mimic the bioactive compounds present in breast milk. Different prebiotic mixtures of galactooligosaccharides (GOS) and fructooligosaccharides (FOS) are applied in an infant’s formula for the bifidogenic effect (71–74). Furthermore, commercially available HMO 2′-fucosyllactose (2′FL) and lacto-N-neotetraose (LNnT) were recently introduced. In consideration of the availability and recent introduction of products with HMO on the market, few studies evaluated the effect of formula with HMO on an infant’s gut microbiome. To date, the evidence suggests that even partial formula feeding in the first days after birth negatively influences the gut microbial composition (32).

### Potential Effect on Firmicutes and Verrucomicrobia

Studies included in our systematic review underlined an increased abundance of Firmicutes. The seeding distinction after C-section favors the growth and colonization of *Enterococcus*, *Clostridia*, or *Veillonella*. These organisms opportunistically take advantage of the depleted taxa and successfully outcompete other bacteria. Interestingly, if we consider the presence of breastfeeding in the first month of a CS infant’s life, no alterations were observed in Clostridiales taxa. Concerning the reported increase in Firmicutes, five of 13 studies employing a broad range of molecular techniques did not prove any significant differences in other taxa than Bacteroides.

The missing contact with maternal vaginal microbiota during birth may be a possible reason for the higher abundance of some genera of the Firmicutes phylum. Studies investigating the role of CS on newborn gut microbiota development often discuss the acquisition of *Lactobacillus* (75, 76). Our systematic review did not show any significant reduction of *Lactobacillus* in CS breastfed infants. This lends support to findings that exclusive breastfeeding is positively associated with the abundance of *Lactobacillus* taxa (77, 78) and that *Lactobacillus* species from breast milk quickly restore their abundance in infants’ gut microbiota after CS.

Furthermore, one of the components of breast milk microbiota is the genus *Akkermansia* belonging to the phylum Verrucomicrobia. Both Tonon et al. (43) and Korpela et al. (40) describe their increased abundance in the gut microbiota of exclusively breastfed infants in secretory mothers. They also suggest that the higher abundance of *Akkermansia* and *Bacteroides* in CS infants of secretory mothers potentially decreases the risk of allergies.

Nevertheless, regarding the consistent significant differences in Bacteroides, other taxa belonging to Firmicutes, Proteobacteria, and Verrucomicrobia, and their changes in abundance are not supported by more than one study within the given timepoint, except for the increase of *Enterococcus* and *Clostridia* supported by two studies.

### Limitations and Strengths

After the systematic literature search, 14 studies stratified breastfeeding status within CS delivery. However, the categorization of feeding practices is not uniform. Some studies classify infants as breastfed if this practice dominates. Other studies strictly distinguished between exclusive and partial breastfeeding. Moreover, the status of breastfeeding is evaluated according to the exact feeding practice at the moment of the sample collection. However, the history and use of formula, most importantly in the first days after birth, are not considered. Notably, the most recent studies observed that the gut microbiota profile of mix-fed infants resembles more formula-fed infants. Even small interventions in breastfeeding may affect the gut microbiota development in the first days of neonatal life (26, 32). The number of studies on formula-fed participants is inadequate, and some included studies presenting negligible sample sizes limit the statistical power. This again emphasizes the importance to record feeding habits and uniform the categorization into exclusive breastfeeding, partial breastfeeding, and exclusive formula feeding when evaluating microbiota development. An adequate number of studies could support the changes in feeding practices after C-section and in the neonatal ward, where formula feeding is a common practice.

An additional limitation is the lack of published data from microbiome analysis that prevent our intention of comparing alpha or beta diversity and conducting any meta-analysis. Moreover, studies differ in chosen sequencing platforms and targeted hypervariable regions of the 16S rRNA gene. Some studies underline the use of universal primers and their underestimation of Bifidobacteria abundance (33).

The strengths of our systematic review are the strict inclusion criteria. Critically, only studies with a control group of vaginally delivered breastfed infants were included. Essential components of the review are the data that combine the information about the number of enrolled infants, mode of delivery, and type of diet. Moreover, our systematic review included some studies with gut microbiome profiles of vaginally delivered infants after ATB treatment. These results emphasize the detrimental effect of ATB treatment, regardless of the delivery mode. Furthermore, we considered the heterogeneity in feeding classification and distinguished between exclusively breastfed, partially breastfed, and non-breastfed infants. To evaluate the dynamics of gut microbiota development in breastfed infants, the results were divided according to the time sampling and were compared to the gut microbiota of formula-fed infants.

Finally, with the increasing incidence of C-section delivery worldwide, our presented data help to characterize the microbial imprint of C-section in breastfed infants. Our results demonstrate the benefit of breastfeeding, which can modify IAP-induced intestinal microbiota changes. However, further research is needed to evaluate diet-induced changes in an infant’s microbiota and also the impact of IAP administration.

## Summary

Unique changes are evident in CS breastfed infants after C-section in gut microbiota development over 3 months after birth. Breastfeeding does not demonstrate the ability to restore the depletion of *Bacteroides* after C-section with affiliated administration of IAP. This in turn may be reflected in the increase in Firmicutes abundance. Furthermore, breastfeeding in CS infants showed a positive influence on Bifidobacteria, which dominate in the healthy gut microbiota of VD breastfed infants and are very sensitive to IAP.

## Data Availability Statement

The original contributions presented in this study are included in the article/Supplementary Material, further inquiries can be directed to the corresponding author.

## Author Contributions

EP participated in literature search, data extraction, and quality assessment. EP wrote the manuscript and designed the figures. IK participated in literature search, data extraction, quality assessment, and reviewed the manuscript. VT supervised and reviewed the manuscript for clarity. All authors contributed to the article and approved the submitted version.

## Conflict of Interest

The authors declare that the research was conducted in the absence of any commercial or financial relationships that could be construed as a potential conflict of interest.

## Publisher’s Note

All claims expressed in this article are solely those of the authors and do not necessarily represent those of their affiliated organizations, or those of the publisher, the editors and the reviewers. Any product that may be evaluated in this article, or claim that may be made by its manufacturer, is not guaranteed or endorsed by the publisher.

## Supplementary Material

Supplementary Table 1 Quality assessment.
