# Supplementary material for: Neonatal Diet and Gut Microbiome Development After C-Section During the First Three Months After Birth: A Systematic Review
Source: Front Nutr. 2022 Jul 26;9:941549. doi: 10.3389/fnut.2022.941549 (PMC9364824; doi:10.3389/fnut.2022.941549)
Supplement: Supplementary Table 1 — Quality assessment. [file Table_1.pdf]

| Autor | Selection | | | | Comparability | Outcome | | |
|---|---|---|---|---|---|---|---|---|
| | 1. representativnes of the exposed cohort | 2. selection of the non exposed cohort | 3. ascertainment of exposure | 4. demonstration that outcome of interest was not present at start of study | 1. comparability of cohorts on the basis of the design or analysis | 1. assessment of outcome | 2. was follow-up long enough for outcomes to occur | 3. adequacy of follow up cohorts |
| Tonon (2021) | * | * | * | * | * | * | | |
| Akagawa (2019) | * | * | * | * | * | * | | |
| Fehr (2020) | | * | * | * | ** | * | | |
| González (2021) | * | * | * | * | * | * | | |
| Brumbaugh (2016) | * | * | * | * | * | * | * | * |
| Chen (2021) | * | * | * | * | ** | * | | |
| Guo (2020) | | * | * | * | * | * | * | * |
| Bokulich (2016) | | * | * | * | ** | * | * | * |
| Madan (2016) | * | * | * | * | * | * | | |
| Hill (2017) | * | * | * | * | * | * | | |
| Korpela (2018) | * | * | * | * | * | * | | |
| Jakobsson | | * | * | * | * | * | * | * |
| Azad (2016) | * | * | * | * | ** | * | | |
| Liu (2019) | * | * | * | * | * | * | | |
